# A validation study of mentees’ views of mentors’ cultural diversity awareness behaviors (CDA race/ethnicity behaviors scale)

**DOI:** 10.1017/cts.2025.10109

**Published:** 2025-07-28

**Authors:** Krystle P. Cobian, Jayashri Srinivasan

**Affiliations:** 1 Geffen School of Medicine, University of California, Los Angeles, USA; 2 School of Education and Information Studies, University of California, Los Angeles, USA

**Keywords:** Validation study, stemm training, cultural diversity awareness, mentoring scale, Enhance Diversity Study

## Abstract

**Introduction:**

Mentorship is a key focus area of efforts to promote a robust biomedical workforce. A growing body of research suggests that mentors’ cultural diversity awareness is an important factor in effective mentoring that can support mentees’ academic and professional outcomes. Less is known about the psychometric properties of scales assessing cultural diversity awareness (CDA). Further, researchers may not have space in surveys to measure all aspects of CDA. We examine the factorial structure and measurement invariance of the CDA Race/Ethnicity (R/E) Behaviors Scale, a subscale from an originally developed CDA Scale, using a large diverse undergraduate sample enrolled in higher education institutions funded by the National Institutes of Health BUilding Infrastructure Leading to Diversity (BUILD) Initiative.

**Methods:**

The CDA R/E Behaviors Scale measures mentees’ perceptions of a mentor’s behaviors concerning cultural diversity awareness. Using a diverse national sample of over 4,000 undergraduate mentees, we examined dimensionality, reliability coefficients, and measurement invariance of the scale.

**Results:**

We found high reliability among the items with a Cronbach’s alpha of 0.91 and Omega of 0.91. Items indicated a unidimensional scale and we found differential item-functioning (DIF) with respect to gender, but no DIF across race/ethnicity.

**Conclusion:**

This study lends additional evidence to support construct validity of the CDA R/E Behaviors Scale, a subscale of the originally developed CDA Scale. Our study supports its use in future research for other science and medical training programs, and contributes tools to program evaluation efforts aimed at improving culturally responsive mentorship throughout the scientific career pathway.

## Introduction

Within the science, technology, engineering, mathematics, and medicine (STEMM) training and workforce environment, mentorship – the professional alliance between individuals to support mutual career growth and development – is a central area of focus in efforts to improve training and retain career interests in STEMM fields [1]. Employing an approach to mentoring that acknowledges and integrates mentees’ experiences and multiple facets of their identity is particularly important for individuals from underrepresented groups in STEMM [2,3] who encounter structural barriers that inhibit their access, retention, and success throughout their education and training [4]. A majority of STEMM mentees do not endorse a racially color-blind approach to mentoring, but rather an approach that is cognizant and responsive to issues of race and racism that can negatively impact mentees [5], and ultimately disrupt their STEMM trajectories. Research suggests that individuals without access to culturally responsive mentoring can experience identity interference and/or identity conflict [1]. Further, a lack of cultural diversity awareness (CDA) can contribute to harmful misconceptions that can have material negative consequences for mentees from racially marginalized groups, including departure from the STEMM training pathway and workforce.

While implementation and evaluation of mentoring models that focus on cultural diversity [6,7] are necessary for improving efforts for persons excluded because of their ethnicity or race (PEERs) [8], there is a need to develop and validate new scales that aim to measure attitudes, behaviors, and perceptions [9] related to the growing body of research on cultural diversity in mentoring relationships. Recently, there has been increased attention to quantitatively assessing cultural diversity awareness – a person’s ability to recognize one’s own culturally shaped beliefs, perceptions, and judgments, and awareness of those differences and similarities in other individuals [6]. Researchers conducted a pilot study to develop and validate the CDA Race/Ethnicity (R/E) Scale [6] which aims to measure beliefs and behaviors regarding racial diversity and inclusion. Since its development, the full scale has been used to examine perceptions from both mentees and mentors [6,10]. However, early studies utilized the full CDA R/E Scale, which cannot always be accommodated into surveys with limited space for additional items. Further, developers of the scale have called for additional validation efforts to ensure the items are functioning with a more diverse pool of participants. Thus, “to facilitate the development of new, valid, and reliable scales, and to help improve existing ones” [9] [p. 2], further validation of the subscales within the full CDA R/E Scale is warranted to ensure its use produces meaningful and trustworthy data for future STEMM training evaluation and research.

Scholars addressing national aims to support a robust STEMM workforce for all Americans continue to work toward improving reliability and validity of key scales that are important for evaluating STEMM intervention programs. Improving reliability and validity of scales, such as CDA, is critical for the interpretability and the generalizability of constructs that multiple researchers utilize to measure the impact of interventions for students from underrepresented groups in the scientific workforce [11,12]. Measures of CDA are important for understanding mentorship and students’ perceptions of mentorship relationships in STEMM, and ultimately examining long-term outcomes on academic and career trajectories.

The Enhance Diversity Study (EDS) [13], implemented by the Coordination and Evaluation Center (CEC) at the University of California, Los Angeles, incorporated CDA into its broader evaluation of capacity-building efforts to broaden participation in biomedical research training at higher education institutions. Participating institutions in the study include 10 programs at 11 geographically diverse, primarily undergraduate institutions across the United States that were recipients of the BUilding Infrastructure Leading to Diversity (BUILD) award [14]. Funded by the National Institutes of Health (NIH) Diversity Program Consortium over a 10-year period (2014–2024), the BUILD sites developed approaches intended to determine effective strategies for engaging and retaining students from diverse backgrounds in biomedical research, and for preparing students to become future contributors to the NIH-funded research enterprise [13]. The EDS collected survey data from undergraduates at each institution, yielding a diverse pool of students and a large data source for the validation of newly developed scales. Needing to balance survey length while also not sacrificing reliability and validity, the Behaviors subscale (called the CDA R/E Behaviors Scale throughout the rest of the manuscript) was selected for use in consultation with the National Research Mentoring Network and original developers of the full CDA scale [6].

The key focus of this study is to (a) examine the factorial structure and reliability coefficients of the CDA R/E Behaviors Scale and (b) employ an Item Response Theory (IRT) framework to assess the item parameters and measurement invariance across race/ethnicity and gender. In addition to validating the behaviors sub-scale of the original CDA scale, we also utilized a diverse sample of undergraduates from institutions across the United States to examine how the CDA R/E Behaviors Scale performed across different subgroups within this population. This study provides evidence to support construct validity of the behaviors subscale, which includes analysis of reliability coefficients, fitting an item response theory model, and assessing measurement invariance across subgroups. We provide recommendations and implications for individuals interested in measuring mentorship in scientific and medical training environments [11,12].

## Materials and methods

### Data source and sample

Students enrolled at the BUILD institutions were invited to complete the Student Annual Follow-up Survey (SAFS), developed by CEC and administered annually between 2017 and 2023 as part of the EDS [15]. The SAFS examined students’ perceptions and views on various educational and career goals, as well as experiences during their time in college. We draw from the 2019 SAFS dataset across the 10 BUILD sites. The sample includes 4,977 undergraduate students who responded “yes” to having a faculty member whom they considered a primary mentor. In the SAFS, a mentor was defined as “someone who provides guidance, assistance, and encouragement on professional and academic issues” and “can be either someone who is more experienced (or senior) than you or someone who is at an educational or professional level similar to you.”

### Missing data and final sample

In terms of missing data, we deleted all cases wherein students had missing responses across all the five items. There were 1,237 missing cases, reducing the analytic sample to 3,740 students. To conduct factor analysis across the items and to fit item response theory models to the items, it is necessary to have complete cases. For gender and race/ethnicity, we conducted a listwise deletion. This further reduced our sample to 3,084 students. We did not conduct any tests to examine missing data mechanisms, since we believe it would not be appropriate impute gender and race/ethnicity for students. The demographic distribution of our final analytic sample is as follows: 64% identified as women; 32% identified as Latine, 18% identified as Black and/or African American, 20% identified as Asian, 23% identified as White, 6% identified as American Indian or Alaska Native (AI/AN), and less than 1% students identified as Native Hawaiian or Pacific Islander (NHPI). In terms of students’ majors, almost 51% were enrolled in natural sciences, 13% were in biomedical social sciences, and 36% were non-biomedical majors.

### Cultural diversity awareness race/ethnicity (CDA R/E) behaviors scale

Byars-Winston and colleagues initially developed and validated the full version of the CDA R/E faculty and matched mentee scales [6]. The original scale for faculty mentors consisted of four subscales, each measuring a dimension of CDA as it relates to race and ethnicity: (1) attitudes, (2) behaviors, (3) confidence to enact CDA, and (4) motivation to enact CDA. Of these, the *behaviors subscale* was used for the mentee version of the scale on the SAFS. For the Behaviors scale, the undergraduate mentees were asked to rate their mentor’s behaviors with respect to CDA. The CDA R/E Behaviors Scale consists of five items designed to “assess the extent to which mentors incorporated CDA practices into their research mentoring relationships” [6] [p. 3]. While the subscale captures behaviors, it may not fully account for mentors’ internal attitudes or biases that also influence mentee experience. Responses to these items are on a 5-point Likert scale ranging from “never” to “all the time” (see Table 1).

**Table 1.** Cultural diversity awareness race/ethnicity behaviors scale for mentees

Item Number	Item
1	My mentor created opportunities for me to bring up issues of race/ethnicity as they arose.
2	My mentor encouraged me to think about how the research related to my own lived experience.
3	My mentor was willing to discuss race and ethnicity, even if it may have been uncomfortable for him/her.
4	My mentor raised the topic of race/ethnicity in our research mentoring relationship when it was relevant.
5	My mentor approached the topic of race/ethnicity with me in a respectful manner.

Note: Scale for items is Never = 1; Rarely = 2; Sometimes = 3; Frequently = 4; All of the time = 5; I choose not to answer = 9.

### Analyses

#### Dimensionality

To investigate the psychometric properties, we first examined the response percentages across the five items. Prior to fitting the factor analytic models to assess the dimensionality, we conducted the Kaiser–Meyer–Olkin (KMO) test. The KMO test is a statistical measure to determine the suitability of data for factor analysis, and it measures the sampling adequacy for each variable in the model. Next, to examine the dimensionality, we split the dataset into two halves and used a cross-validation approach [16]. We conducted an exploratory factor analysis (EFA) with a varimax rotation with the first half of the dataset and cross-validated whether the factor structure held by conducting a confirmatory factor analysis (CFA) with the second half of the dataset. We used the standardized root mean square residual (SRMSR), the Tucker-Lewis Index (TLI), and the root mean square error of approximation (RMSEA) index to assess model fit. The TLI is expected to be greater than 0.90 and RMSEA should be less than 0.05, indicating a good model fit [17].

#### Reliability coefficients

Next, we examined the polychoric inter-item correlations. After establishing unidimensionality we examined the reliability coefficients. While Cronbach’s alpha has historically been the most popular statistics for assessing reliability, recently other statistics such as the omega hierarchical (ω_h_) [18,19] have been utilized as a more appropriate measure, especially when there are multiple items that capture a common underlying construct.

#### IRT parameters and measurement invariance

Finally, we fitted a graded-response model to obtain the item parameters for the five items with a 5-point Likert scale. Item Response Theory (IRT) is a model-based measurement approach in which individuals’ probability of item responses is modeled as a function of the individual’s ability or latent trait, for example, students’ perceptions of the CDA R/E practices of their mentors [20]. IRT is important in comparison to classical test theory as it provides latent trait estimates of the observed responses reported by students, which are spread out as a distribution in comparison to a single summary score [21]. Rather than using a summed score or average score of a scale, which can lead to biased results, IRT emphasizes students’ response patterns. IRT enables fairer and more valid measures for a diverse group of students which is vital in large-scale surveys. IRT is also advantageous as it enables researchers to utilize the same items with a different sample and retain the statistical properties, such as the discrimination parameter and the respondents’ scores that represent the latent trait on a particular construct, which are independent of the test items that were administered [22].

We examined model fit using the M2 function in the mirt package in R, which is specifically designed to assess the fit of item response models for ordinal data. We examined the M2-based root mean square error of approximation (RMSEA), the standardized root mean square residual (SRMSR), and comparative fit index (CFI). We also examined the local independence, which assesses the relationship between pairs of items after controlling for the common latent trait [23]. We used the Yen’s Q_3_ statistic [24] which is considered suitable for polytomous data. Next, we conducted a differential item-functioning (DIF) analysis to examine whether students responded differently to the items based on their race/ethnicity and gender. If multiple items exhibit DIF, this may indicate the scale’s inability to utilize a common metric across different subgroups. To assess DIF, we conducted Wald tests with Benjamini-Hochberg adjustment. All the analyses were conducted using mirt [25] and psycModel [26] packages in R software [27].

## Results

### Descriptive analysis

**Figure 1.**
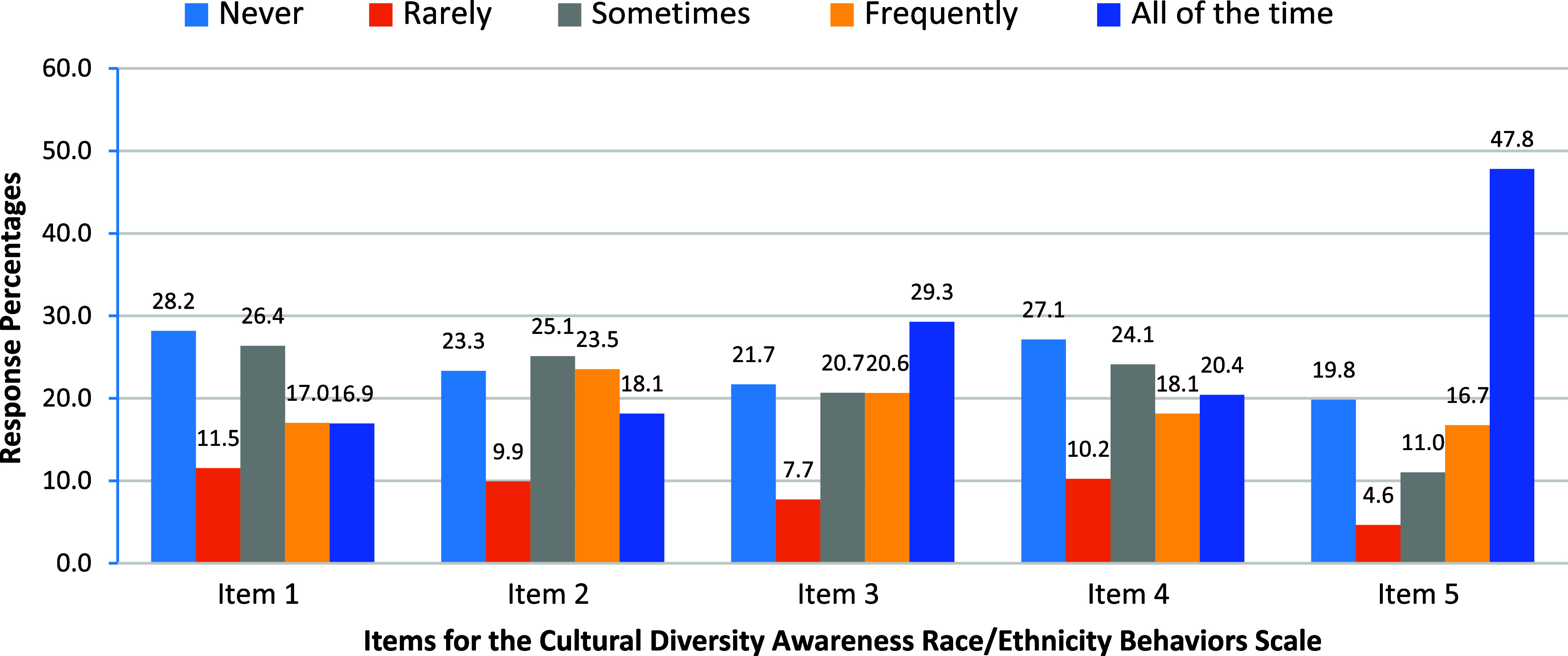
Bar chart of the response percentages to cultural diversity awareness race/ethnicity behaviors scale items (*N* = 3,740).

To examine the psychometric properties of the CDA R/E Behaviors Scale, we assessed the response percentages across the five items (Figure 1). With respect to a mentees’ primary mentors creating opportunities to bring up issues of race/ethnicity (Item 1); 40% of mentees responded “never” or “rarely,” whereas 34% noted that this occurred “frequently” or “all the time.” When mentees were asked to what extent their mentors encouraged them to think about how their research related to the mentee’s lived experience (Item 2), 33% responded never/rarely, compared to a larger proportion (42%) who responded frequently/all of the time. Whether a mentor was willing to discuss race and/or ethnicity, even if it may have been uncomfortable (Item 3), 29% of the mentees responded never/rarely; half of mentees responded that they never discussed race and/or ethnicity. Regarding the extent to which a mentor raised the topic of race/ethnicity in the research mentoring relationship when it was relevant (Item 4), 38% reported never/rarely, and almost the same proportion (39%) reported that this occurred frequently/all of the time. Last, with respect to their mentors approaching the topic of race/ethnicity in a respectful manner (Item 5), one out of four students responded never/rarely, compared to almost two out of three students (65%) who reported that their mentors approached race/ethnicity respectfully either frequently or all the time.

### Dimensionality

The overall KMO for the data was 0.88. The higher the KMO-value, the more suited the data are to factor analysis. Table 2 presents the factor loadings for the two sets of samples. EFA results of the first subsample showed satisfactory fit to the data. The model fit statistics indicated a X^2^ (5) = 121.62, *p* < 0.001, CFI = 0.98, RMSEA = 0.12, SRMSR = 0.03, and TLI = 0.95. The recommended RMSEA is usually below 0.05. However, since all the other model fit statistics are satisfactory, we concluded that the overall model fit is adequate. The factor loading was the smallest for Item 2 (*mentor encouraged thinking about how the research related to one’s own lived experience*). CFA results for the second subsample also indicated good model fit. The model fit statistics were RMSEA = 0.10 (90% confidence interval of 0.084 and 0.12), SRMSR = 0.02, and TLI = 0.98. The CFA results suggest high construct validity as results indicate a single-factor structure.

**Table 2.** Factor loading for cultural diversity awareness race/ethnicity behaviors scale items (*N* = 3,084)

	Exploratory Factor Analysis (EFA) Factor loadings for the first half of the of sample	Confirmatory Factor Analysis (CFA) Factor loadings for second half of the sample
Item 1	0.84	0.80
Item 2	0.67	0.63
Item 3	0.91	0.89
Item 4	0.89	0.88
Item 5	0.81	0.81

### Reliability coefficients

Next, we examined the polychoric inter-item correlations, which ranged from 0.60 (for Items 2 and 5) to 0.86 (Items 3 and 4). Cronbach’s alpha for the reliability coefficient was 0.91. An acceptable range for Cronbach’s alpha to assess the psychometric quality of the scales is usually between 0.80 and 0.95 [28]. The omega hierarchical (ω_h_) reliability coefficient was 0.91. Additionally, the IRT-based marginal reliability [29] for a unidimensional scale was 0.88. The Average Variance Extracted (AVE) is the measure of the variance captured by the construct in relation to the amount of variance because of measurement error. Here, the AVE was 0.675. This means that, on average, 67.5% of the variation in the CDA R/E Behaviors Scale is explained by the five items.

### IRT parameters and measurement invariance

A graded-response model was fit the to the five Likert-scale items. The model fit statistics indicated that model fit was adequate. The RMSEA value = 0.082 (95% CI [0.069, .095]) and SRMSR value = 0.029 suggest that data fit the model reasonably well using suggested cut-off values of RMSEA ≤ 0.06 and SRMSR ≤ .08. The TLI was 0.98, which is above the recommended 0.95 cut-off value. Next, we examined local dependency and focused on item pairs which had correlations higher than 0.3 [30]. Items 3 and 4 had a higher correlation (−0.482) than expected, indicating that this pair of items warrants further investigation. It is worth noting that it is often difficulty to fully satisfy the local independence assumption with a unidimensional scale, making it vital to examine all aspects of overall model and item characteristics [30,31].

Next, we examined the the IRT parameters to help us understand the characteristics of the items (Table 3). The slope or the discrimination parameter is a measure of how well an item differentiates responses of participants with different levels of the latent trait. In other words, larger values, or steeper slopes, are better at differentiating on the latent trait. The latent trait in this context is the latent CDA R/E Behaviors measure, which is the students’ perceptions of the CDA R/E practices of their mentors.

**Table 3.** Item response theory parameters for cultural diversity awareness race/ethnicity behaviors scale

	Slope Parameter	Thresholds or Location Parameters			
	a	b1	b2	b3	b4
Item 1	3.34	−0.63	−0.28	0.42	1.02
Item 2	1.75	−1.00	−0.58	0.26	1.22
Item 3	5.09	−0.76	−0.46	0.07	0.56
Item 4	4.57	−0.60	−0.29	0.29	0.82
Item 5	3.27	−0.88	−0.64	−0.25	0.16

A slope also can be interpreted as an indicator of the strength of a relationship between an item and latent trait, with higher slope values corresponding to stronger relationships. The slope parameter (*a*) ranges from a minimum of 1.75 to a maximum of 5.09. For example, Item 3 was the most discriminating items with a slope estimate of 5.09, while Item 2 was the least discriminating item with a slope estimate of 1.75. Note that a higher slope discrimination value also relates to the inter-item correlations.

The threshold values or location parameters (*b1* to *b4* in Table 3) can be interpreted as the value of a latent trait that corresponds to a 50% probability of responding at or above that location on an item. *b1* is the cut-off point of responding to category 1 vs categories 2, 3, 4, or 5; *b2* is the cut-off point of responding to categories 1 or 2 vs. categories 3, 4, or 5; *b3* is the cut-off point of responding to categories 1, 2, or 3 vs. categories 4 or 5, and so on. For example, for Item 4, a student who has a CDA R/E score of –0.60 has a 50–50 chance of selecting category 1; a student with a latent score –0.29 has a 50–50 chance of selecting category 1 or 2 vs. category 3, 4, or 5. Results indicate difficulty differentiating between students at the mean, or one to two standard deviations above or below the mean.

The location parameters indicate that the responses did not cover a wide range of the latent trait. That is, they ranged from –1 to 1, with students responding to lowest (*never*) and highest (*all the time*) categories. For example, for Item 5, we observed that a student with a threshold value of −0.25 (*b3*) is below the mean on the latent trait and yet responds to categories 3 or 4. This indicates that for this item the range is limited, and not spread out covering the entire range of the latent trait. In other words, we found that the students are mostly endorsing the highest categories, which is also seen in Figure 1, wherein nearly 47% students respond “all the time” to Item 5. In this case, a higher score indicates a mentee’s positive perception of their mentor’s CDA behaviors, meaning that the mentees are more likely to select a more positive rating on the items that make up this scale.

We generated category response curves (CRC), item information curves (IIC), and test information function (TIF), with standard error of measurement (SEM) (see Supplementary Materials). A plot of the test information versus the latent trait can indicate the quality of the items across the latent trait, and the amount of information (the height on the *y*-axis, high or low) reflects how well the items discriminate. The IIC for Items 3 and 4 provided the most information across all levels of the latent trait. That is, there is a smaller SEM associated with these items. Item 2 provided the least information to the CDA R/E Behaviors Scale and expand on possible explanations for this result in the discussion. The overall TIF was found to be high with a low SEM indicating precise CDA R/E latent trait estimates.

Regarding measurement invariance of the scale, we examined students’ responses to the CDA R/E items to whether there were any group differences by gender and race/ethnicity. First, with respect to gender (Table 4), we found DIF for Item 3 (*Mentor willing to discuss race and ethnicity, even if it may have been uncomfortable*) (*p* < 0.05). In other words, for this item the IRT parameters differed across women (*a* = 4.972) and men (*a* = 6.591). With respect to race/ ethnicity, we did not find any DIF across the items for the four groups: Asian, Black/African American, Latine, and White. We expand on the implication of these results in the discussion.

**Table 4.** Differential item-functioning (DIF) analysis across gender

	Gender		Race/Ethnicity	
Items	Wald Statistic	Adjusted *p*-values	Wald Statistic	Adjusted *p*-values
Item 1	2.91	0.22	2.734	0.491
Item 2	0.63	0.62	0.434	0.726
Item 3	6.41	0.05	0.094	0.759
Item 4	0.08	0.77	0.305	0.726
Item 5	0.46	0.62	1.607	0.512

### Discussion & implications

This validation study of the CDA R/E Behaviors Scale utilizes a national sample of undergraduate students from 10 BUILD awarded sites nationwide who were part of the EDS sponsored by NIH and is significant for researchers who are interested in using the five–item Behaviors Scale (CDA R/E Behaviors Scale) when the full CDA scale may not be feasible to include in surveys. We examined the factorial structure and measurement invariance of the CDA R/E Behaviors Scale. Overall, we found that the scale is performing well for a diverse sample of undergraduates across the U.S. Examining dimensionality, factor loading was the smallest for Item 2 (*Mentor encouraged thinking about how the research related to one’s own lived experience*), similar to Byars-Winston and Butz [6] results for mentees on this item. This result may be due to the wording of the item in relation to the other items, which primarily focused on race/ethnicity.

Next, while we found local dependency between items pairs 3 and 4, we believe these items could be related as mentees may perceive that their mentors raised the topic of race/ethnicity when it is relevant, and “even in cases when they were uncomfortable to talk about it.” It is crucial to capture all aspects of the test characteristics, such as over model fit, the CRC, IIC, DIF to get a complete picture of the items and the scale. We found the IRT-based marginal reliability to be 0.88, which speaks to the overall test while taking into account the distribution of the underlying latent trait. DIF analyses not only shows how students are interpreting the items but can also guide us to dig deeper into subgroups who might be responding to items differently. To our knowledge, this is the first study to examine the measurement invariance of the CDA R/E Behaviors Scale across gender. In examining whether there were any differences in how groups responded to items in the scale, we found differences between men and women for Item 3, “my mentor was willing to discuss race and ethnicity even if it may be uncomfortable.” While the overall scale might be functioning well, the DIF results for item 3 implies that the probability of responding to this item will differ among men and women undergraduates at the same level of CDA R/E behavior trait based on how they interpret the item. Men undergraduates are more likely to respond to the higher categories, indicating they feel more strongly that their mentors are willing to discuss issues of race and ethnicity compared to women undergraduates. Some research suggests group differences in attitudes toward cultural sensitivity, with women and racial/ethnic minority medical students scoring higher on a cultural sensitivity index compared to their male or White counterparts (Lee & Coulehan, 2006.) It could be that if women, on average, are more attuned to cultural issues, they may be more critical of their mentor’s willingness to discuss race and ethnicity. This finding warrants further investigation such as, ensuring that the item is interpreted accurately by men and women either by conducting focus groups or by reviewing the items with them via in-depth interviews to better understand why we see these results. These results also allude to the need to include IRT and measurement invariance checks as a routine during item development and refinement processes.

We found no DIF in how students responded to the items by race/ethnicity. In this case, it would be important to test whether individuals were responding to a scale about race and ethnicity based on their own racial/ethnic identity. Further, because of the larger sample with both STEMM and non-STEM majors compared to the original validation study of the full CDA R/E Scale, this study lends additional evidence that mentees are perceiving the items in the CDA R/E Behaviors Scale similarly. This validation study provides additional confidence to the use of the CDA R/E Behaviors Scale for future research and program evaluation examining culturally responsive mentorship.

The results also contribute to the understanding of race and/or ethnicity in faculty mentoring of undergraduate mentees. Data from the EDS used to validate the scale also serves as documentation of undergraduate faculty mentor behaviors from the perspective of undergraduate mentees at the BUILD sites in 2019. The large proportion of students indicating that faculty mentors frequently or always engaged in CDA behaviors suggests that there is a relatively high level of CDA among faculty at the BUILD institutions – many of which are minority serving and research-rising institutions. For context, several BUILD sites implemented practices that supported integration of students’ social identities and scientific identities [3]. Further, several sites specifically implemented faculty mentor training, with some sites incorporating specific training that emphasized CDA [32] and race and racism [33], and addressed stereotype threat [34]. BUILD-trained faculty reported more engagement and confidence with mentoring compared to non-BUILD faculty [35]. While connecting these initiatives to mentorship outcomes is outside the current scope, these analyses indicate that further research is warranted at institutions that implement faculty mentorship trainings at scale. Further, it will be important to see how ratings by race, ethnicity, and gender, since the absence of DIF findings does not necessarily mean the average group scores are equivalent.

### Limitations

This study has several limitations. First, the original CDA scale includes four subscales, and was designed to pair the primary mentor’s and mentee’s scores for study. The EDS did not collect data to pair the CDA scores of each primary mentor for the student respondents. Next, we were only able to conduct DIF analysis for race/ethnicity across four race/ethnicity groups due to small sample sizes for NHPI and AI/AN groups. This points to the challenge of validating scales regarding issues of race and ethnicity and the structural barriers that continue to exclude students from racially marginalized groups from higher education [36] and result in their erasure from higher education research findings. Third, the response option, “Never” could be misinterpreted by survey participants. For example, a participant might select “never” to mean that issues of race/ethnicity did not arise, rather than indicating that the mentor did not create opportunities to discuss issues of race. Further cognitive interviews can help determine whether response options need to be adjusted to clarify their interpretation. Fourth, potential response bias may exist given the nature of participant self-reporting on a mentor’s behaviors. Last, the BUILD awards provided funding to implement and test student and faculty-level initiatives, which included faculty mentor training [14]. Determining which undergraduates had a primary mentor who participated in BUILD mentor training was outside of the scope of the study, but this factor is important to comprehensive analysis and description of this unique sample of undergraduates from the BUILD institutions.

### Recommendations for use and future research

There are several implications for use of the CDA R/E Behaviors Scale, one aspect of the original CDA R/E full scale [6] for the clinical and translational science field. By confirming that the five behavioral items can be used together to compile a picture of the latent variable – students’ perceptions of mentors’ CDA regarding race/ethnicity – these results open possibilities for researchers who are limited on survey data space but want to measure CDA. The CDA R/E Behaviors Scale can be added to common measures used in consortia or partnerships examining postsecondary science and biomedical training outcomes. Our results indicate that the scale is unidimensional with a high reliability coefficient, and absence of DIF across items establish that the scale can be used as a predictor or an outcome in regression analyses to produce robust empirical evidence. Researchers can interpret differences in responses as true differences, and not because of different interpretations of the items based on gender or race. Additionally, even though the scale consists of just five items, the overall test information function provides adequate amount of information along the trait continuum. In the middle of the trait distribution the test provides more information with a small standard error indicating higher reliability of the scale.

Future work can measure changes in CDA for mentors or mentees at the BUILD sites, or serve as a comparison data set for other institutions interested in using the CDA R/E Behaviors Scale. Future use might also consider using gender-neutral language in the wording of questions and/or examining whether adjustments need to be made to the response options.

Future research can examine CDA as both an individual measure and for use as an organizational measure of campus climate [3] for culturally aware mentorship. Additionally, future work can also examine and validate modified versions of the CDA scale that address other social identities (socio-economic status, disability, etc.) as well as intersectionality [37]. A longitudinal study of students can provide an indication of changes over time in mentees’ evaluations of their mentors, as well as examining the impact of CDA on outcomes such as matriculation into graduate study and/or retention in the STEMM workforce. Lastly, IRT should be utilized alongside an underlying theory of a scale of interest and while working with item content experts. A good understanding of the latent trait that is being studied (in this case, CDA) is vital, along with an awareness of how students are responding or endorsing items that make up the latent trait [22].

This work can be expanded to fit the broader context of scientific and medical training, where there are often multiple-PI research projects and a range of trainees at various stages in their careers. Future research can examine how the CDA R/E Behaviors Scale functions for graduate students, postdoctoral scholars, and early-career faculty researchers. Alternatively, this scale might also be applied beyond scientific research careers, such as the field of medicine with medical students, interns, residents, attending physicians, and senior mentors. Regardless of field, the researchers can decide whether to use the mentee or mentor scale depending on the focus of the study and/or interventions. However, more research is needed to see how the Behaviors scale for mentee respondents works for a non-undergraduate population. It is also important to note that the original scale was intended to be used by pairing and comparing mentor and mentee responses. Future research and practice can implement and test how mentors/mentees respond depending on the mentor and mentee career stage.

## Conclusion

Developing the knowledge and skills to address issues related to race and racism can be supportive to all trainees regardless of their own racial/ethnic identity and the racial/ethnic identity of the mentor [1,38]. Considering the large body of research on the harms of race-neutral approaches to education and mentorship [8,38–40], particularly for PEERs, it is critical to examine how both perceptions and behaviors are impacting mentees. Neville and colleagues [41] propose that color-blind racial ideology consists of two interrelated domains: color-evasion (the denial of racial differences) and power-evasion (denial of racism via emphasis on the existence of equal opportunities) [41]. Efforts to measure the extent to which faculty are moving toward or away from color-evasive and power-evasive perspectives can provide a tangible sense of perceptions and behaviors related to race and racism.

It is important to empirically examine role of culturally aware mentoring in advancing goals of supporting a talented STEMM workforce, considering that mentorship is one of the primary evidence-based mechanisms for talent development in STEMM training environments and there is a robust body of research on the benefits of cultural awareness skills and behaviors. Additionally, recent calls to end “DEI” efforts must not conflate notions of merit in STEMM career advancement [42] with attitudes and practices that “do not see race” at all in training and workplace environments. Scales such as the CDA R/E Behaviors Scale that capture mentors’ capacity to be responsive to racism can help track the progress of programs that aim to impact faculty behaviors. While attitudes of mentors with respect to cultural diversity are important, mentees’ perceptions of a mentor’s behaviors indicate the extent to which faculty behaviors are received by the individuals that they wish to support via mentoring relationships. Mentees’ perceptions of CDA may impact long-term outcomes, such as academic persistence or career advancement. Thus, the CDA R/E Behaviors Scale is important for assessing how a mentee perceives a mentor’s actions with respect to awareness of issues related to race and ethnicity.

## Supporting information

10.1017/cts.2025.10109.sm001 Cobian and Srinivasan supplementary material Cobian and Srinivasan supplementary material

## Supplementary material

The supplementary material for this article can be found at https://doi.org/10.1017/cts.2025.10109
