# Supplementary material for: A validation study of mentees’ views of mentors’ cultural diversity awareness behaviors (CDA race/ethnicity behaviors scale)
Source: J Clin Transl Sci. 2025 Jul 28;10(1):e42. doi: 10.1017/cts.2025.10109 (PMC12975626; doi:10.1017/cts.2025.10109)
Supplement: Cobian and Srinivasan supplementary material [file S205986612510109Xsup001.docx]

**Supplementary Material**

**Category Response Curves**

The category and item characteristics curves provide researchers with a better picture of the items and their relationship to the latent trait, especially when we are studying psychological traits^30^. Figure S1 depicts the category response curves (CRCs), which is the relationship between how students responded to the various response categories across the five items. These plots help in visualizing the probability of endorsing a specific response category and the cut-offs between two curves is the threshold value (*b*). For items 1 and 4, students selected category 2, *rarely,* (depicted by the less peaked P2 curve in Figure 1) much less in comparison to other categories, such as category 3 or category 4 (depicted by the peaked P3 and P4 curves in Figure 1). For item 5, all students around mean (latent trait, 𝜃) of zero responded to category 5, *all the time.* Students above the mean have a much higher probability of selecting “5” than any other category. Half of the students responded to the highest category, which makes it harder to differentiate between respondents at the mean compared to respondents one or two standard deviations above or below the mean.


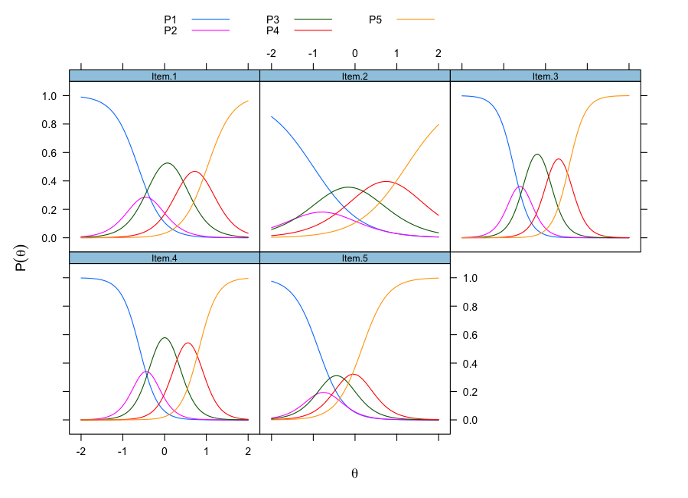


Figure S1: Category Response Curves (CRCs) Depicting the Probabilities of Responding to the Specific Categories in an Item’s Response Scale

Note: P1 corresponds to Item 1, P2 = Item 2, P3= Item 3, P4 = Item 4, and P5= Item 5 from the CDA Race/Ethnicity Behaviors Scale

**Item Information Curves**

The item information curves (IIC) (Figure S2) depict how precisely each item measures the CDA R/E latent trait at various levels of the scale. The distribution of the response percentages to the items indicated that the students responded to all the categories, instead of responding to only highest categories, which is often seen in survey responses. The CDA R/E Behaviors Scale consists of five items designed to “assess the extent to which mentors incorporated cultural diversity awareness practices into their research mentoring relationships.”^5^ These items together provide students’ perceptions of the CDA race/ ethnicity practices of their mentors. Item 3 (*my mentor was willing to discuss race and ethnicity, even if it may have been uncomfortable for him/her*) and item 4 (*my mentor raised the topic of race/ethnicity in our research mentoring relationship when it was relevant*) provide the most information across all levels of the latent trait. That is, there is a smaller standard error of measurement associated with these items. On the other hand, items 1 and 5 provide slightly less information; and item 5 (m*y mentor approached the topic of race/ethnicity with me in a respectful manner*) is more useful at the lower latent trait of the scale in comparison to item 1 (*my mentor created opportunities for me to bring up issues of race/ethnicity as they arose*)*.*

Finally, item 2 (*my mentor encouraged me to think about how the research related to my own lived experience*) provides the least information to the scale which relates to the lowest discrimination (or slope) among all the items.


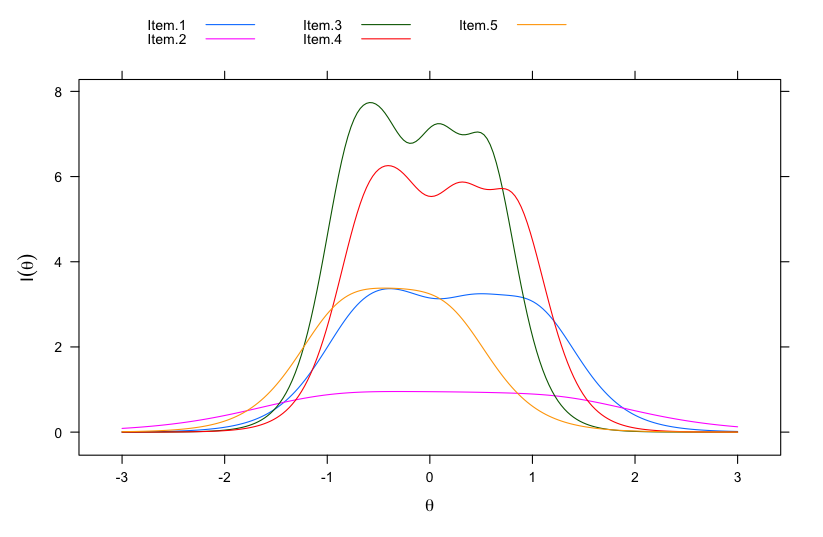


Figure S2: Item Information Curves (IICs) for the Cultural Diversity Awareness Race/Ethnicity Behaviors Scale

**Test Information Function**

Figure S3 shows the Test Information Function (TIF; solid line) and the Standard Error of Measurement (SEM; dashed line) for the scale. TIF includes the information of all items across the range of the latent trait distribution (𝜃), thus providing overall information of the scale. SEM is the inverse of the information, whereby as the information increases, the standard error decreases. The TIF under the general graded response model can be interpreted similarly to that of the general dichotomous response model^1^.

The CDA R/E scale provides good information in the middle of the trait distribution (𝜃) and the amount of information provided by the scale decreases as we move to the extremes of the trait distribution. Furthermore, we found a high distribution for the information function which is expected in most cases for a reliable scale. The SEM (denoted by dashed red line in Figure S3) is relatively low in comparison to the information (y-axis) across the range of the trait distribution from –2 to +2. In other words, the higher the information along the CDA R/E scale we find lower standard errors which result in precise θ estimates. Additionally, we found the IRT-based marginal reliability for a unidimensional scale to be 0.88, lending further confidence about the reliability of the CDA R/E scale.


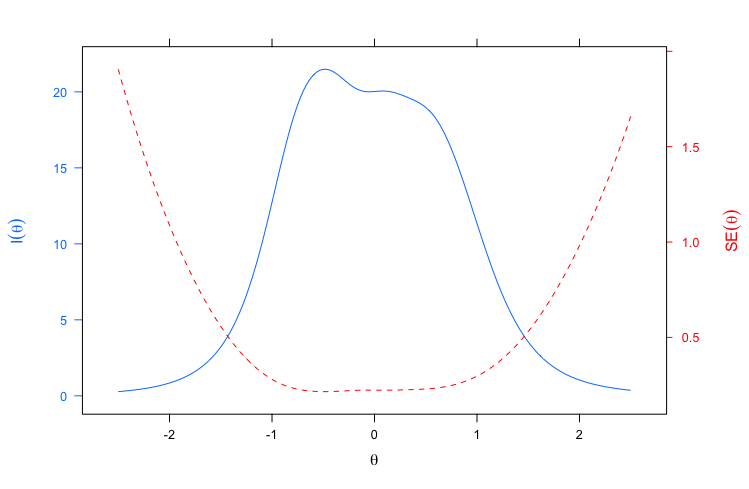


Figure S3: Test Information Function and the Standard Error for the overall Cultural Diversity Awareness Race/Ethnicity Behaviors Scale

**Reference**

1. Samejima F. The general graded response model. *Handbook of polytomous item response theory models*. Routledge; 2011:77-107.
